# Catheter-induced left main dissection during valve-in-valve TAVI: a rare complication of coronary protection strategy

**DOI:** 10.1093/ehjcr/ytaf584

**Published:** 2025-12-24

**Authors:** Alessandro Comis, Sebastiano Immè, Claudia Tamburino, Corrado Tamburino

**Affiliations:** Cardiology Department, Policlinico Centro Cuore Morgagni, Via della resistenza 30, Pedara 09053, Italy; Cardiology Department, Policlinico Centro Cuore Morgagni, Via della resistenza 30, Pedara 09053, Italy; Cardiology Department, Policlinico Centro Cuore Morgagni, Via della resistenza 30, Pedara 09053, Italy; Cardiology Department, Policlinico Centro Cuore Morgagni, Via della resistenza 30, Pedara 09053, Italy

## Case report

**Figure 1:**
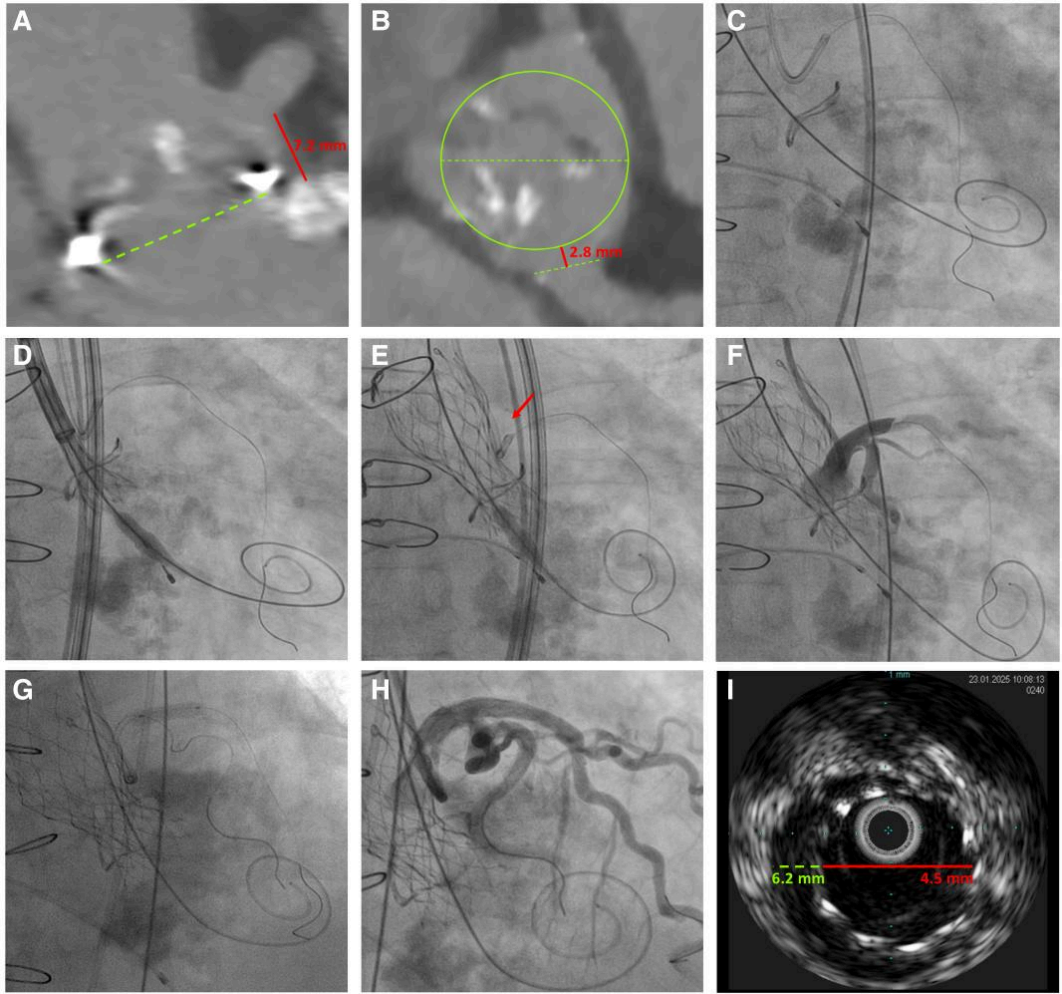
Central illustration. (*A* to *B*) preprocedural CT scan (multiplanar reconstruction). (*A*) Coaxial bioprothesis diameter (green dotted line) and LM ostium height (red line). (*B*) Plane above aortic anulus showing a simulation of transcatheter aortic valve perimeter (green circle) with small valve to coronary distance (red line). (*C*) wire positioned to the distal LDA. (*D*) Initiation valve deployment. (*E*) Valve released causing catheter displacement (red arrow). (*F*) Post implantation coronary angiography showing LM dissection. (*G*) Bailout stenting. (*H*) Restoration of coronary flow. (*I*) IVUS to optimize stent implantation. CT = computer tomography; IVUS = intravascular ultrasound; LAD = left anterior descending; LM = left main.

An 83-year-old woman with dysfunction of a MitroFlow No. 19 aortic bioprosthesis was referred for transcatheter aortic ViV. Given her advanced age and high surgical risk, she was deemed inoperable. Preprocedural computed tomography revealed a low left coronary ostium (7.2 mm) and inadequate valve-to-coronary distance (2.8 mm) (*Figure 1A* and *B*), indicating a high risk of coronary obstruction. To mitigate this risk, a coronary protection strategy was employed via a radial approach, placing a wire in the distal left anterior descending (LAD) artery (*Figure 1C*). An Evolut PRO + 23 mm valve (Medtronic) was partially deployed (*Figure 1D*), and aortography confirmed preserved flow in the left main (LM) and distal coronaries and the valve was released (*Figure 1E*). However, post-deployment angiography revealed an anterograde LM dissection with LAD occlusion (*Figure 1F*), likely due to catheter displacement during valve release. The patient developed severe chest pain and a new left bundle branch block (LBBB). Urgent intervention was performed with a second coronary wire placed in the left circumflex artery. Pre-dilatation with a semi-compliant balloon failed to restore flow, necessitating bailout stenting from the LM to the proximal LAD with three drug-eluting stents (*Figure 1G*). TIMI-3 flow was achieved (*Figure 1H*), resolving both LBBB and symptoms. Intravascular ultrasound was used to optimize stent deployment (*Figure 1I*). The patient remained asymptomatic the following day, with elevated high-sensitivity troponin (1083 ng/dL) but no wall motion abnormalities on echocardiography.

Catheter-induced coronary dissection is a rare but life-threatening complication.^1^ To our knowledge, this is the first reported case occurring during coronary protection for ViV implantation. To reduce the risk of such complications during coronary protection, it is advisable to use the chimney technique: pull back the guide catheter into the ascending aorta leaving closed stent into coronary artery or use of a guide extension catheter and placement the stent inside to avoid deep catheter intubation.^2^ Alternatively, to prevent coronary occlusion a leaflet modification could have been performed using techniques such as BASILICA or UNICORN.^3^

The patient has given written consent to the use of his/her clinical data for educational and research purposes according to COPE guidelines.

The data underlying this article will be shared on reasonable request to the corresponding author.

## Data Availability

The data underlying this article will be shared on reasonable request to the corresponding author.

## Notes

**Funding:** No funding was received for the preparation and publication of this manuscript.
